# Contact-induced Andreev bound states in normal-metal/superconductor planar junctions

**DOI:** 10.1093/nsr/nwaf105

**Published:** 2025-03-21

**Authors:** Beilin Wang, Linhai Guo, Guopei Ying, Wenbu Duan, Jian Li, Changgan Zeng

**Affiliations:** CAS Key Laboratory of Strongly-Coupled Quantum Matter Physics, and Department of Physics, University of Science and Technology of China, Hefei 230026, China; International Center for Quantum Design of Functional Materials (ICQD), Hefei National Research Center for Physical Sciences at the Microscale, University of Science and Technology of China, Hefei 230026, China; Hefei National Laboratory, University of Science and Technology of China, Hefei 230088, China; CAS Key Laboratory of Strongly-Coupled Quantum Matter Physics, and Department of Physics, University of Science and Technology of China, Hefei 230026, China; International Center for Quantum Design of Functional Materials (ICQD), Hefei National Research Center for Physical Sciences at the Microscale, University of Science and Technology of China, Hefei 230026, China; Hefei National Laboratory, University of Science and Technology of China, Hefei 230088, China; CAS Key Laboratory of Strongly-Coupled Quantum Matter Physics, and Department of Physics, University of Science and Technology of China, Hefei 230026, China; International Center for Quantum Design of Functional Materials (ICQD), Hefei National Research Center for Physical Sciences at the Microscale, University of Science and Technology of China, Hefei 230026, China; Hefei National Laboratory, University of Science and Technology of China, Hefei 230088, China; Institute of Natural Sciences, Westlake Institute for Advanced Study, Hangzhou 310024, China; School of Science, Westlake University, Hangzhou 310024, China; Institute of Natural Sciences, Westlake Institute for Advanced Study, Hangzhou 310024, China; School of Science, Westlake University, Hangzhou 310024, China; CAS Key Laboratory of Strongly-Coupled Quantum Matter Physics, and Department of Physics, University of Science and Technology of China, Hefei 230026, China; International Center for Quantum Design of Functional Materials (ICQD), Hefei National Research Center for Physical Sciences at the Microscale, University of Science and Technology of China, Hefei 230026, China; Hefei National Laboratory, University of Science and Technology of China, Hefei 230088, China

**Keywords:** tunneling spectroscopy, Andreev bound state, superconductivity, planar junction, oxide interface

## Abstract

Tunneling spectroscopy is a powerful tool for investigating the pairing mechanism of superconductors. However, the use of planar tunneling junctions to characterize Andreev bound states associated with unconventional superconductivity remains largely unexplored. Here, we report tunneling spectroscopy studies of normal-metal/superconductor planar junctions, leveraging the newly discovered LaAlO_3_/KTaO_3_(111) superconducting interface. We observe distinct spectroscopic behaviors for weak and strong tunneling barriers, controlled by the thickness of the LaAlO_3_: when the barrier is weak, the metallic contact couples strongly with the interface superconductor, resulting in pronounced double peaks within the superconducting gap; when the barrier is strong, the in-gap peaks diminish and a softened full superconducting gap profile emerges. These observations, supported by theoretical calculations, pinpoint the presence of contact-induced Andreev bound states in planar junctions. This not only reveals the possibility of *p*-wave superconductivity at the LaAlO_3_/KTaO_3_ interface but also offers a universal approach to identifying the most sought-after superconducting states.

## Teaser

This study investigates tunneling spectroscopy in Ti/ LaAlO3/KTaO3 junctions, revealing potential p-wave superconductivity at the LaAlO3/KTaO3 interface and offering a universal approach to identifying the most sought-after superconducting states.

## INTRODUCTION

Unconventional superconductivity [1–4] has long been a wonderland in condensed matter research where fascinating physics emerges from the interplay of strong electron correlation, spin-orbit coupling, band topology, etc. Among the various types of unconventional superconductivity, each defined by an unusual form of superconducting order parameter, the *p*-wave one in 2D is particularly alluring because of its close connection to topological phases of matter [5,6] as well as non-abelian quasiparticle excitations [7,8]. Tunneling spectroscopy serves as an effective means for probing the pairing mechanism of superconductors [9,10], offering many candidates for *d*-wave or *p*-wave superconductivity by detecting Andreev bound states (ABSs) [9,11–17]. Nonetheless, existing research has rarely addressed the characterization of ABSs in planar junctions with out-of-plane tunneling into unconventional superconductors.

**Figure 1.**
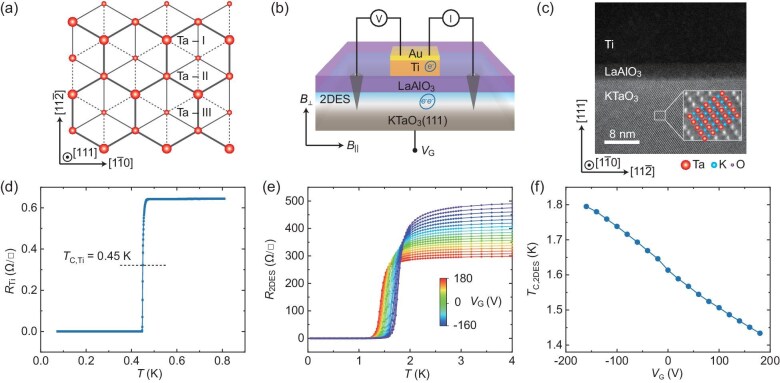
Characterization of the Ti/LAO/KTO(111) device. (a) Distribution of Ta^5+^ ions along the [111] crystal axis of the KTO(111) surface. Ta^5+^ ions are shown with progressively smaller sizes in the three adjacent (111) planes, which are labeled as Ta-I, Ta-II and Ta-III, respectively. (b) Schematic of a Ti/LAO/KTO device for quasi-four-point tunneling measurements with the LAO layer serving as the barrier. (c) STEM image of Ti/LAO/KTO viewed along the [1$\bar 1$0] crystal axis. (d) Temperature dependence of the Ti electrode sheet resistance. (e) Temperature dependence of the 2DES sheet resistance at different gate voltage *V*_G_. The data were taken in Sample #1, and the applied current in these measurements flows along the [11$\bar 2$] crystal axis. (f) Superconducting transition temperature of the 2DES extracted from (e) as a function of *V*_G_.

Recently, superconductivity with a critical temperature (*T*_C_) reaching up to ∼2 K has been discovered in two-dimensional electron systems (2DES) at the (111)-oriented KTaO_3_(KTO)-based heterointerfaces, such as EuO/KTO [18] and LaAlO_3_/KTO (LAO/KTO) [18,19]. This 2DES originates from the 5*d* orbitals of the Ta ions, which form a buckled honeycomb lattice (see Fig. 1a). The Ta 5*d* electrons feature remarkably strong spin-orbit coupling (SOC), ∼0.4 eV in energy scale [20], which has been deemed favorable for the formation of unconventional superconductivity [21,22]. Indeed, the parallel critical magnetic field for the superconducting 2DES has been reported to exceed the Pauli paramagnetic limit, hinting at possible *p*-wave paring states [18]. Additionally, in-plane 2-fold symmetric oscillations observed in the upper critical magnetic field also suggest the presence of *p*-wave pairing at KTO-based interfaces [23]. So far, however, the studies of this superconducting 2DES have been limited to in-plane electronic transport [18,19], leaving the superconducting order parameter and pairing mechanism unclear.

In this work, we performed tunneling conductance spectroscopy measurements in normal-metal/insulator/superconductor (N/I/S) planar junctions composed of Ti/LAO/KTO structures. These measurements exhibit characteristic in-gap features arising from ABSs that are prominent when the tunneling barrier is weak but suppressed when it is strong, which is in full agreement with theoretical expectations and model calculations for this setup, signifying *p*-wave superconductivity.

## RESULTS AND DISCUSSION

The N/I/S tunneling junctions in our experiment, illustrated in Fig. 1b, are composed of Ti as the metallic electrode and LAO/KTO with the LAO layers serving as the insulating tunnel barrier (see Methods, and Figs S1–S3). In the case of a weak barrier, the LAO film was usually grown to a thickness of 3.2 nm, close to the minimum thickness required to induce 2DES at the LAO/KTO interface [24]. The scanning transmission electron microscopy (STEM) image (Fig. 1c) and the chemical map (Fig. S2) show the junction with sharp interfaces, where the LAO tunnel barrier is uniformly amorphous. This implies that the growth temperature of 620 °C is not sufficient to crystallize LAO on this particular KTO substrate.

The Ti electrode was characterized by its sheet resistance as a function of temperature (*T*), shown in Fig. 1d, which indicates its superconducting critical temperature (*T*_C, Ti_) to be 0.45 K. In this work, we define *T*_C_ as the temperature at which the resistance reaches half of its normal-state value. The temperature dependence of the 2DES sheet resistance for different gate voltages (*V*_G_) is presented in Fig. 1e, where superconducting transitions with clear *V*_G_-dependence can be observed. Specifically, the superconducting critical temperature of the 2DES (*T*_C,2DES_) can be tuned from 1.43 K to 1.80 K as *V*_G_ varies from 180 V to −160 V (see Fig. 1f). Note that we did not observe the dome-shaped *T*_C_–*V*_G_ dependence reported in a previous work [19] due to the fact that the carrier density of our samples, estimated to be ∼1.8 × 10^14^ cm^−2^ (see Fig. S4), exceeds the optimal doping density (∼8 × 10^13^ cm^−2^), thereby placing our samples in the overdoped regime.

Following previous studies [25–29], we first assessed the validity of tunneling in our Ti/2DES junctions (configured with quasi-four-point measurements as shown in Fig. 1b). Specifically, in the normal state at *T* = 3 K, we demonstrated that the measured resistance mainly comes from the Ti/2DES junction, and importantly, the conductance of the Ti/2DES junctions increases with bias voltage, exhibiting prototypical tunneling characteristic [30,31] (see Fig. S3c and Note S1 and Note S2 for details).

**Figure 2.**
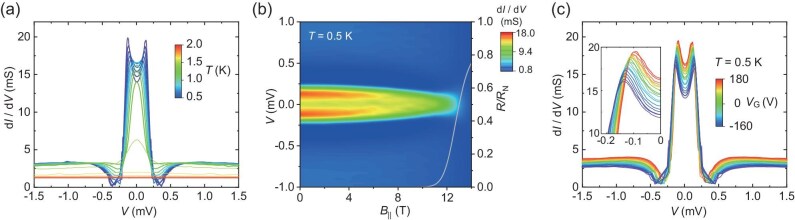
Tunneling spectra of the Ti/LAO/KTO(111) device with *d*_LAO_ = 3.2 nm. (a) Tunneling spectra at different temperatures without an external magnetic field. (b) Differential tunneling conductance as a function of the bias voltage and the parallel magnetic field at *T* = 0.5 K, overlaid with the field-dependent 2DES normalized resistance curve in gray for reference. (c) Tunneling spectra for different gate voltages *V*_G_ at *T* = 0.5 K. Inset: Zoom in on the left peaks. All measurements were performed in Sample #1.

Next, we performed tunneling spectroscopy measurements at a temperature of 0.5 K, between *T*_C, Ti_ and *T*_C,2DES_, where our junctions are well-defined N/I/S tunneling junctions. The measured differential conductance spectra for Sample #1 are shown in Fig. 2a for various temperatures and *V*_G_ = 0 V. In particular, the tunneling spectrum at 0.5 K exhibits pronounced double peaks located at ±0.12 meV and double dips at ±0.37 meV, both symmetric at around zero bias. As temperature increases, these peaks and dips are gradually suppressed and eventually disappear at 1.7 K, where the 2DES turns normal. Similar behaviors can also be observed by applying and gradually increasing the parallel magnetic field, as shown in Fig. 2b. Moreover, when *V*_G_ is varied from 180 V to −160 V, the tunneling spectrum at 0.5 K displays a monotonic shift of the double peaks from ±0.09 meV to ±0.14 meV, and that of the double dips from ±0.30 meV to ±0.44 meV (see Fig. 2c), which agrees with the *V*_G_ dependence of *T*_C,2DES_ presented in Fig. 1f. The measurements conducted on other samples further confirm that the above features are generic in our devices (see Fig. S5).

To explain the observed features, we can first exclude several possibilities. For conventional *s*-wave superconductors [32,33], in-gap differential conductance peaks can occur in the otherwise U-shaped tunneling spectral gaps in a variety of scenarios. Among these scenarios, a major class [28,29,34–40], such as Kondo resonance [28,36] and reflectionless tunneling [29,34], features a single peak centered at zero bias in the absence of an external magnetic field, which clearly does not agree with our observation. One exception where split peaks naturally arise involves tunneling upon the Yu-Shiba-Rusinov (YSR) states [41–43] induced by magnetic impurities [37]. In this scenario, however, the pair of peaks generally carry significantly different spectral weights in a local tunneling measurement [35,38–40] and the peak centers, though symmetric at around zero bias, can split or shift in a strong magnetic field due to the Zeeman effect [39,40]. These expected behaviors for YSR states are not seen in our measurements, shown in Fig. 2b, where the symmetrically weighted double peaks remain immobile with an increasing parallel magnetic field up to its critical strength. As a cross check, we also performed an EDS elemental mapping of the interface and found no discernable presence of magnetic elements such as Fe, Co, Cr and Mn with 0.1% resolution. Such evidence excludes conventional YSR states as an explanation for our main experimental results.

An alternative explanation that merits consideration is the critical current effect within the metallic transport regime [44]. This effect could produce features similar to what we have observed when combined with conventional Andreev conductance. To address this scenario, we have thoroughly analyzed the normal-state transport characteristics of our junctions and examined the positions of the conductance peaks and dips in the spectra (see Note S1 for details). Collectively, this scenario does not likely apply to our case. Therefore, we will focus on exploring intrinsic mechanisms beyond conventional *s*-wave superconductivity for the 2DES in our LAO/KTO setup.

Indeed, ABSs that do not rely on any magnetic ingredient to occur in unconventional superconductors [9,10] can be a strong candidate. It is well-known that nonmagnetic scattering is able to break Cooper pairs in *p*-wave or *d*-wave superconductors and lead to in-gap ABSs analogous to the YSR states in *s*-wave superconductors [37]. Such quasiparticle states have been observed routinely as either single or split in-gap conductance peaks in tunneling spectroscopy data on cuprate superconductors [9,11], and possibly as double conductance peaks and double dips in in-plane tunnel junctions of superconducting Sr_2_RuO_4_ [12] (noting that the superconducting pairing symmetry of Sr_2_RuO_4_ is still under debate [45]) or point contacts in topological materials, such as 3D Dirac semimetal Cd_3_As_2_ [13] and Weyl semimetal TaAs [14]. Surprisingly, however, to our knowledge, characterization of ABSs in a planar junction involving out-of-plane tunneling into an unconventional superconductor has been barely discussed in the literature. It is this scenario that we proceed to examine in theory and establish its pertinence to our experimental observations.

**Figure 3.**
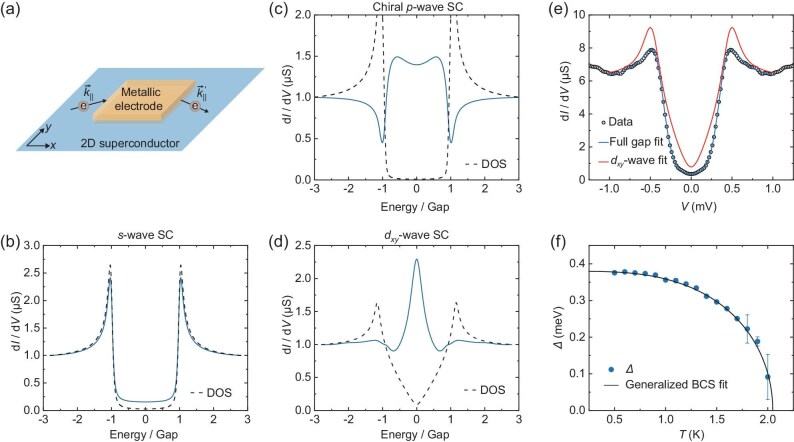
Contact effect in the tunneling spectrum. (a) Schematic of an out-of-plane N/B/S planar junction, where the metallic electrode is in contact with a part of the underlying superconductor through a tunneling barrier. Depending on the barrier thickness, or the metal-superconductor coupling strength, electrons in the 2D superconductor are scattered at different rates, causing contrasted features in the tunneling spectrum for an unconventional superconductor. (b–d) Calculated tunneling spectra (blue lines, normalized by the 2DES normal state value), when the metal-superconductor coupling is strong, for generic *s*-wave, chiral *p*-wave and *d_xy_*-wave superconductors, respectively. The normalized DOS for a pristine superconductor in each case is also plotted (in black dashed lines) for reference. (e) Tunneling spectrum, plotted as black circles, of the Ti/LAO/KTO(111) device with *d*_LAO_ = 4 nm (in the weak metal-superconductor coupling regime) at *T* = 0.5 K. The data here is symmetrized from the raw data for Sample #4. The blue and red lines are the Dynes model fits by using full gap and *d_xy_*-wave gap functions, respectively. (f) Temperature dependence of $\Delta$ extracted from the above fitting with a full gap function, where the error bar indicates the standard deviation of each fitting. The black line shows a further fit of this temperature dependence by the generalized BCS model with *T*_C_ = 2.05 K and 2$\Delta$(*T* = 0)/*k*_B_*T*_C_ = 4.3.

As a generic origin of ABSs, we focus on the inherent scattering of electrons in planar junctions, caused by the necessary contact of the electrode with a certain region of the sample (see Fig. 3a). The effect of such contact can be accounted for theoretically by including a self-energy term ${{\mathrm{\Sigma }}_c}$ in the superconductor Green's function, $G = {( {G_0^{ - 1} - {{\mathrm{\Sigma }}_c}} )^{ - 1}}$, where ${G_0}$ stands for the mean-field Green's function for the pristine superconductor without contact. When impurities are neglected, ${G_0}$ is diagonal with respect to momentum in the Nambu basis [46]. The self-energy ${{\mathrm{\Sigma }}_c}$, by contrast, is generally non-diagonal, signifying scattering in the momentum space due to the presence of the contact. Consider, for example, the electrode to be modeled as a particle-in-a-box in its transverse dimensions (parallel to the junction interface), the contact self-energy ${{\mathrm{\Sigma }}_c}( {{{\mathord{\buildrel{{\ \rightharpoonup}} \over k} }_\parallel },\mathord{\buildrel{{\rightharpoonup}} \over k} {^{\prime}_\parallel }} ) \propto \mathop \prod \nolimits_{i = x,y} \,\,[ {\delta ( {{k_i} - k_i^{\prime}} ) - \delta ( {{k_i} + k_i^{\prime}} )} ]$ (see Note S3) with the in-plane momentum $\mathord{\buildrel{{\rightharpoonup}} \over k} _\parallel ^{( ^{\prime} )} = ( {k_x^{( ^{\prime} )},k_y^{( ^{\prime} )}} )$, which clearly introduces backscattering in either component of the momentum via the $\delta ( {{k_i} + k_i^{\prime}} )$ term. Consequently, pairing channels associated with different momenta become mixed. This mixing has no significant effect in *s*-wave superconductors where the order parameter associated with different pairing channels are identical, in line with the Anderson theorem [47], but results in pair breaking in an unconventional superconductor where by definition the pairing order parameter takes on an anisotropic form. For a *p*-wave superconductor, in particular, the order parameter is an antisymmetric function of momentum, and the contact-induced backscattering can strongly suppress local pairing, leading to in-gap quasiparticle excitations (see Note S3 for details) that can in turn be detected by the tunneling junction.

The heuristic analysis above can be made precise by performing explicit transport simulations and comparing the obtained features with the experimental ones. To that end, we employ a lattice model of the junction illustrated in Fig. 3a and compute the differential conductance by using the scattering matrix for the metallic contact (see Methods for the numerical simulations details). We consider three types of pairing, namely *s*-, *p*- and *d*-wave, for the superconductor, and the main simulation results of these are shown in Fig. 3b–d, respectively. These results exhibit sharp contrast in terms of the qualitative features in differential conductance, especially with reference to the quasiparticle density of states (DOS) in a pristine superconductor that can be obtained from ${G_0}$. In the *s*-wave case, the differential conductance curve follows the DOS and there exists no appreciable in-gap feature (see Fig. 3b); in the *p*-wave case, henceforth using a chiral *p*-wave order parameter to be specific, the differential conductance develops dips near to where the coherence peaks in the DOS sit, as well as pronounced double peaks inside the original, hard DOS gap (see Fig. 3c); in the *d*-wave case, using a *d_xy_*-wave order parameter for demonstration, dips in the differential conductance curve occur at inwardly shifted energy values relative to the coherence peaks, and a single peak occurs at the center of the otherwise V-shaped DOS gap (see Fig. 3d). It is now evident that our experimentally observed features presented in Fig. 2 (see also Fig. S5) agree with the case of *p*-wave superconductivity in the 2DES.

To further scrutinize the *p*-wave interpretation, we note that the tunneling rate between the metal and the LAO/KTO interface in our junctions depends exponentially on the thickness of the LAO film, *d*_LAO_, permitting access to different regimes of the contact effect by varying *d*_LAO_ in our sample preparation. In this regard, the device in Fig. 2, with *d*_LAO_ = 3.2 nm (Sample #1), has a normal-state junction resistance of ∼0.93 kΩ at 3 K. This value is comparable to that reported in previous tunneling studies [25,48,49], and indicates that the device falls into the strong contact-2DES coupling regime where ABSs are effectively induced to present pronounced features in the tunneling spectrum. Likewise, the simulations in Fig. 3b–d are performed (blue lines) in the strong coupling regime with sufficiently large tunneling rates (see Methods for the numerical simulation details).

By opting for other devices, for example, with *d*_LAO_ = 4 nm (Sample #4) and the junction resistance being about 140 kΩ at 3 K (two orders of magnitude larger than the preceding one), we are also able to access the weak coupling regime and observe a differential conductance profile, shown in Fig. 3e, which resembles a softened full superconducting gap. This tunneling spectrum, verified by the simulation of the *p*-wave case in a weakly coupled junction (see Fig. S6), can be fitted remarkably well as broadened DOS for a *p*-wave superconductor, but deviates significantly from a *d*-wave one (see Fig. 3e). By fitting the tunneling spectra at different temperatures with the Dynes formula [50], we obtain the temperature dependence of the full superconducting gap $\Delta$ that is consistent with the generalized BCS theory [32,33,51], with $\Delta$ = 0.38 meV, *T*_C,2DES_ = 2.05 K and 2$\Delta$(*T* = 0)/*k*_B_*T*_C,2DES_ = 4.3 (see Fig. 3f, as well as Note S4 and Fig. S7 for details). Notably, the absence of in-gap differential conductance peaks in the weakly coupled junctions further excludes the relevance of YSR states that exist independent of the contact-2DES coupling [35,37–40], but strongly favors the scenario of the contact-induced ABSs when conjoined with the strong coupling features.

**Figure 4.**
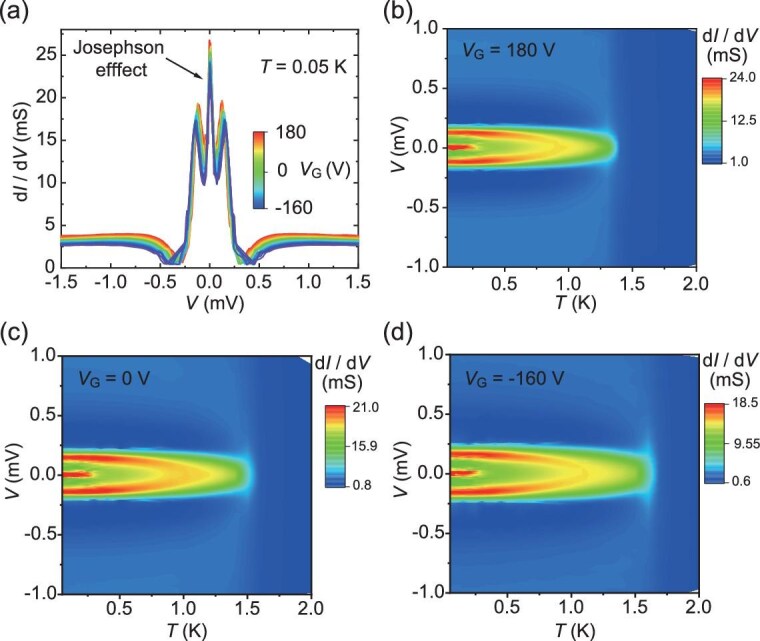
Josephson effect in tunneling spectra at temperature below *T*_C, Ti_. (a) Tunneling spectra for different *V*_G_ at *T* = 0.05 K, where the Ti electrode and the 2DES are both superconducting. (b–d) Temperature-dependent tunneling spectra for *V*_G_ = 180 V, 0 V and −160 V, respectively. All measurements were performed in Sample #1.

We next complement the characterization of our Ti/LAO/KTO junctions by measurements at temperatures lower than the superconducting critical temperature of Ti, *T*_C, Ti_ = 0.45 K (see Fig. 1d), giving our devices the form of Josephson junctions. Specifically, the measured tunneling spectra for different *V*_G_ at *T* = 0.05 K are shown in Fig. 4a. We observe that on top of the main features already seen at 0.5 K for a normal-superconducting junction (cf. Fig. 2), an additional, prominent zero-bias conductance peak emerges in the Josephson junction. Such enhanced differential conductance near zero bias becomes suppressed at ±0.06 meV, which is consistent with the predicted Ti superconducting gap (∼±0.07 meV) from *T*_C, Ti_ = 0.45 K. The temperature-dependent tunneling spectra for *V*_G_ = 180 V, 0 V and −160 V, shown in Fig. 4b–d, respectively, indicate that the zero-bias conductance peaks invariably disappear at temperatures above 0.35 K, which is close to *T*_C, Ti_ and further supports the origin of the zero-bias conductance peaks to be a Josephson supercurrent between the Ti electrode and the 2DES. The zero-bias conductance peak reaches a height ∼8 times that of the normal-state conductance, which may be attributed to thermal or noise-induced fluctuations [52–54].

## CONCLUSION

We have performed systematic tunneling spectroscopy measurements on the Ti/LAO/KTO(111) planar junctions and developed a unified phenomenological theory along with model calculations to characterize the underlying superconducting states. We observe strong evidence suggesting *p*-wave superconductivity in the 2DES at the LAO/KTO interface. To fully understand such an unconventional superconductor requires development of a microscopic theory for this system and is beyond the scope of the present work. Nevertheless, we conjecture that the strong SOC associated with the Ta 5*d* electrons in KTO, which is ∼0.4 eV in contrast to ∼0.02 eV for the Ti 3*d* electrons in SrTiO_3_ [20], plays a key role here. Additionally, the resultant strong SOC in the 2DES may also have facilitated the observed Josephson coupling between the *s*-wave superconductor Ti and the *p*-wave one at the LAO/KTO interface [55], which is another intriguing problem for future study. Overall, our comprehensive investigation of the planar tunneling junctions offers new insights into the search for and characterization of exotic superconductivity in controllable devices.

## METHODS

### Device fabrication

The LAO/KTO samples were prepared by depositing LAO films on KTO(111) single crystalline substrates (MTI Corporation) using the pulsed laser deposition method. Before growth, the substrates were pre-annealed *in situ* in an oxygen pressure of 1 × 10^−4^ mbar at 620°C for ∼30 mins. Please note that the annealed KTO substrate remains insulating. We deposited amorphous LAO thin films from a single-crystalline target under identical temperature and pressure (see Note S5). The thickness of the LAO film was controlled by counting the growth laser pulses. The growth rates calibrated by atomic force microscopy were ∼0.0067 nm/pulse (Fig. S1). In order to obtain a sufficiently small tunneling barrier, the LAO film is usually grown to a thickness of 3.2 nm, close to the minimum thickness required to generate the 2DES [24]. To obtain a sufficiently large tunneling barrier, the LAO film is usually grown to a thickness of 4 nm.

After growth, the LAO/KTO samples were cooled down to room temperature at the growth oxygen pressure. Subsequently, the samples were transferred into a sputtering system. With the home-made Si_3_N_4_ hard mask, 70 nm of Ti (purity 99.995%) was deposited onto the sample surface by DC sputtering, followed by capping with 10 nm of Au (purity 99.99%) to protect the Ti from oxidation. The Ti/Au top electrode has a typical area of 500 μm × 500 μm. Eventually, the Ti/LAO/KTO junction was established, as illustrated in Fig. 1b. The microstructure of the junction was assessed by scanning transmission electron microscopy (STEM), which shows that LAO is homogeneous (Fig. 1c and Fig. S2). To obtain the high-angle annular dark-field (HAADF) image in Fig. 1c, the prepared sample was relatively thin (<100 nm). To obtain the energy dispersive X-ray spectroscopy (EDS) elemental mapping in Fig. S2, the prepared sample was relatively thick (>300 nm).

### Transport measurements

For electric measurements, Au wires were connected to the Ti/Au electrodes with Ag paint, and Al wires were connected to the interfacial 2DES by wire bonding. Then, typical quasi-four-point tunneling measurements were performed with the LAO film as a tunnel barrier (Fig. 1b). To control the transport characteristics electrostatically, Ti/Au film as a gate electrode was deposited on the back side of the KTO substrate. Tunneling spectra were acquired by sourcing current from the top Ti/Au electrode to the right (or left) electrode of the 2DES and measuring the voltage between a second wire on the top electrode and the left (or right) electrode of the 2DES. The differential conductance d*I*/d*V* (*V*) was obtained from the derivative of the *I*-*V* curves of the tunnel junction. The measurements were performed in an Oxford Triton dilution refrigerator and Oxford Instruments ^4^He cryostat.

### Numerical simulations

To simulate transport in our junctions, we employ the standard scattering theory applied to generic models consisting of three parts: superconductor (*SC*), metallic lead (*L*) and tunneling between them (*T*). Thus, the Hamiltonian equation formally reads


(1) \begin{eqnarray*}
H = {H_{SC}} + {H_L} + {H_T}.
\end{eqnarray*}

We assume that the superconductor is of two dimensions and infinite size, and the tunneling between the lead and the superconductor is local. We further assume the metallic lead to be finite-sized but sufficiently large (compared with its Fermi wavelength) in the dimensions parallel to the junction interface and semi-infinite in the dimension perpendicular to the interface. Explicitly, the lattice model Hamiltonian for the superconductor, ${H_{SC}},$ is given by one of the following depending on the pairing form under consideration:


(2a) \begin{eqnarray*}
{H_{sSC}} = \mathop \sum \limits_{\boldsymbol k} \left( {c_{\boldsymbol k \uparrow }^\dagger ,{c_{ - \boldsymbol k \downarrow }}} \right)\left( {{\xi _{\boldsymbol k}}{\tau _z} + {\Delta _s}{\tau _x}} \right)\left( {\begin{array}{@{}*{1}{c}@{}} {{c_{\boldsymbol k \uparrow }}}\\ {c_{ - \boldsymbol k \downarrow }^\dagger } \end{array}} \right),
\end{eqnarray*}

(2b) \begin{eqnarray*}
{H_{pSC}} &=& \mathop \sum \limits_{\boldsymbol k} \left( {c_{\boldsymbol k}^\dagger ,{c_{ - \boldsymbol k}}} \right)\left[ {\xi _{\boldsymbol k}}{\tau _z} + {\Delta _p}\sin \left( {{k_x}a} \right){\tau _x}\right.\\
&&\left. + {\Delta _p}\sin ( {{k_y}a}){\tau _y} \right]\left( {\begin{array}{@{}*{1}{c}@{}} {{c_{\boldsymbol k}}}\\ {c_{ - \boldsymbol k}^\dagger } \end{array}} \right),
\end{eqnarray*}

(2c) \begin{eqnarray*}
{H_{dSC}} &=& \mathop \sum \limits_{\boldsymbol k} \left( {c_{\boldsymbol k \uparrow }^\dagger ,{c_{ - \boldsymbol k \downarrow }}} \right)\left[ {\xi _{\boldsymbol k}}{\tau _z}\right.\\
&&\left.+ {\Delta _{{d_{xy}}}}\sin ( {{k_x}a} )\sin ( {{k_y}a} ){\tau _x} \right]\left( {\begin{array}{@{}*{1}{c}@{}} {{c_{\boldsymbol k \uparrow }}}\\ {c_{ - \boldsymbol k \downarrow }^\dagger } \end{array}} \right),\\
\end{eqnarray*}

where $c_{\boldsymbol k\sigma }^\dagger $/${c_{\boldsymbol k\sigma }}$ are the electron creation/annihilation operators for 2D momentum $\boldsymbol k = ( {{k_x},{k_y}} )$ and spin $\sigma = \uparrow , \downarrow $ (in the *p*-wave case we consider effectively spinless electrons and therefore drop the spin indices); ${\xi _{\boldsymbol k}} = 2t[ {2 - \cos ({k_x}a) - \cos ({k_y}a)} ] - \mu $ is the normal state energy with the lattice constant *a*, the hopping parameter *t*, and the chemical potential $\mu $; ${\Delta _s}$, ${\Delta _p}$ and ${\Delta _{{d_{xy}}}}$ are the *s*-, *p*-, and ${d_{xy}}$-wave order parameters, respectively; ${\tau _{x,y,z}}$ are the Pauli matrices associated with the Nambu basis. The lattice Hamiltonian for the lead is given by


(3) \begin{eqnarray*}
{H_L} = \mathop \sum \limits_{i,\sigma } \left( {6{t_L} - {\mu _L}} \right)\psi _{\boldsymbol i\sigma }^\dagger {\psi _{\boldsymbol i\sigma }} - \mathop \sum \limits_{\left\langle {\boldsymbol {i,j}} \right\rangle ,\sigma } {t_L}\psi _{\boldsymbol i\sigma }^\dagger {\psi _{\boldsymbol j\sigma }},
\end{eqnarray*}

where $\boldsymbol i = ( {{i_x},{i_y},{i_z}} )$ with ${i_x},{i_y} = 1,\,\,2,\,\, \cdots ,\,\,W$ and ${i_z} = 0,\,\,1,\,\,2,\,\, \cdots , + \infty $ is the site index in 3D (similarly for $\boldsymbol j$); $\langle {\boldsymbol {i,j}} \rangle $ stands for a pair of indices for neighboring sites; $\psi _{\boldsymbol i\sigma }^\dagger $/${\psi _{\boldsymbol i\sigma }}$ are the electron creation/annihilation operators in the lead; ${t_L}$ is the hopping parameter and ${\mu _L}$ is the chemical potential. The local tunneling term is given by


(4) \begin{eqnarray*}
{H_T} = \mathop \sum \limits_{{i_x},{i_y},\sigma } V\psi _{\left( {{i_x},{i_y},0} \right)\sigma }^\dagger {c_{\left( {{i_x},{i_y}} \right)\sigma }} + {\mathrm{h}}.{\mathrm{c}}.,
\end{eqnarray*}

where *V* is the coupling parameter between the superconductor and the lead.

To obtain transport properties such as the differential conductance, we first compute the full Green function in the superconductor as $G = {( {G_0^{ - 1} - {{\mathrm{\Sigma }}_c}} )^{ - 1}}$, where ${G_0}$ stands for the mean-field Green's function for the pristine superconductor without connecting to the lead and ${{\mathrm{\Sigma }}_c}$ is the self-energy due to the presence of the lead. Then we compute the scattering matrix for the lead by


(5) \begin{eqnarray*}
S( E) = \mathbb{1} - i{\mathcal{W}^\dagger }G\left( {{E^ + }} \right)\mathcal{W},
\end{eqnarray*}

where $\mathcal{W}$ is the transfer matrix from the lead to the superconductor defined by $\mathcal{W}{\mathcal{W}^\dagger } = i( {{{\mathrm{\Sigma }}_c} - {\mathrm{\Sigma }}_c^\dagger } )$. The zero-temperature differential conductance is then obtained from the scattering matrix by


(6) \begin{eqnarray*}
{\left. {\frac{{dI}}{{dV}}} \right|_{eV = E}} &=& \frac{{{e^2}}}{h}{\mathrm{Tr}}\left[ {\mathbb{1} - {S_{NR}}{{\left( E \right)}^\dagger }{S_{NR}}}\left( E \right)\right.\\
&&\left. {+ {S_{AR}}{{\left( E \right)}^\dagger }{S_{AR}}\left( E \right)} \right],
\end{eqnarray*}

where ${S_{NR}}$ and ${S_{AR}}$ are the normal reflection and Andreev reflection blocks in the scattering matrix, respectively.

For the numerical data presented in Fig. 3b–d, we have computed ${G_0}$ by taking a $100 \times 100$ mesh grid in the ***k***-space for the superconductor, and ${{\mathrm{\Sigma }}_c}$ for a $30 \times 30$ transverse section size (that is, $W = 30$) at zero energy by using Eq. (S7) in Note S3. The rest of the parameters are scaled by *t* (set to 1), and are given in Table S1. We note that the parameters here are not necessarily realistic but instead chosen to diminish the finite-size effect suffered in numerical methods. Additionally, we emphasize that the primary goal of the simulations is to corroborate and highlight the core mechanism of contact-induced ABSs and to demonstrate how a comparison with experimental observations can be used to identify possible pairing forms. In this sense, we do not suggest the pairing order parameter to be precisely the isotropic chiral *p*-wave one assumed in Eq. (S2b). Instead, we stress that our experimental observations can be consistent with other pairing forms involving *p*-wave superconductivity such as an anisotropic chiral *p*-wave or a mixed *s* + *p_x_* order parameter, both of which can explain the observed 2-fold symmetry in the upper critical in-plane field [23].

## Supplementary Material

nwaf105_Supplemental_File
