# Supplementary material for: Contact-induced Andreev bound states in normal-metal/superconductor planar junctions
Source: Natl Sci Rev. 2025 Mar 21;12(6):nwaf105. doi: 10.1093/nsr/nwaf105 (PMC12153723; doi:10.1093/nsr/nwaf105)
Supplement: nwaf105_Supplemental_File [file nwaf105_supplemental_file.docx]

Supplementary Information for

**Contact-induced Andreev bound states in normal-metal/superconductor planar junctions**

Beilin Wang^1,2,3^†, Linhai Guo^1,2,3^†, Guopei Ying^1,2,3^, Wenbu Duan^4,5^, Jian Li^4,5^*, and Changgan Zeng^1,2,3^*

^1^*CAS Key Laboratory of Strongly-Coupled Quantum Matter Physics, and Department of Physics, University of Science and Technology of China, Hefei, China.*

^2^*International Center for Quantum Design of Functional Materials (ICQD), Hefei National Research Center for Physical Sciences at the Microscale, University of Science and Technology of China, Hefei, China.*

^3^*Hefei National Laboratory, University of Science and Technology of China, Hefei, China.*

^4^*Institute of Natural Sciences, Westlake Institute for Advanced Study, Hangzhou, Zhejiang, China.*

^5^*School of Science, Westlake University, Hangzhou, Zhejiang, China.*

†These authors contributed equally to this work.

*Correspondence and requests for materials should be addressed to C. Z. (cgzeng@ustc.edu.cn) and J. L. (lijian@westlake.edu.cn).

**Contents**

1. Supplementary Note
2. Figures and Table
3. References
4. **Supplementary Note**

**Supplementary Note 1. Discussion on alternative explanation of the double conductance peaks and double dips.**

The double conductance peaks and double dips are routinely seen in the metallic regime of transport [1]. However, this mechanism is unlikely to apply to our results based on four facts:

1. In general, for normal junctions in the metallic regime, the conductance (*G* = d*I*/d*V*) decreases with increasing bias voltage (*V*) due to an increase in the inelastic scattering rate of electrons at higher energies [2-4]. In sharp contrast, in the standard tunneling regime, *G* always increases with *V* because of the inelastic tunneling effect opens additional conduction channels [5]. This feature has been widely recognized as a fingerprint of the tunneling effect [3-6]. The structure of our device is Ti/LAO/KTO, with the two-dimensional system (2DES) situated at the LAO/KTO interface. At *T* = 3K, when both Ti and the 2EDS are in the normal metal state, the d*I*/d*V*-*V* curve (see Supplementary Fig. 3c) distinctly shows that the conductance increases with increasing bias voltage. This clearly indicates that the transport operates dominantly in the tunneling regime rather than in the metallic regime, which is further supported by our estimation below. Moreover, our junction resistances are comparable to those reported in the previous tunneling studies [6-8].
2. Suppose the Ti/2DES junction consists of a complex resistance network. Within this network, local resistances can be captured by Sharvin’s formula, Wexler’s formula, or Maxwell’s formula. This configuration suggests the existence of multiple point-contact channels operating across various metallic transport regimes (ballistic, intermediate, thermal). Notably, each channel operates exclusively within one of these regimes because the transport regime of the point-contact is determined solely by its contact radius [9]. It follows that these channels with distinct transport regimes should be considered as being parallel. This complex resistance network can thus be described by a straightforward and rational model, allowing us to estimate the upper bound of the contact area from experimental data as follows:

The Ti/2DES junction conductance ($1/R_{jun}$) is expected to be contributed by parallel channels of Sharvin resistance ($R_{sh}$), Maxwell resistance ($R_{m}$), and Wexler resistance ($R_{w}$), thus is expressed as: $\frac{1}{R_{jun}}=\sum_{i} \frac{1}{R_{sh,i}}+\sum_{j} \frac{1}{R_{w,j}}+\sum_{k} \frac{1}{R_{m,k}}$, where $R_{sh,i}=\frac{2R_{q}}{k_{F}^{2}{a_{i}}^{2}},$ $R_{w,j}=\frac{2R_{q}}{k_{F}^{2}{b_{j}}^{2}}+\frac{\rho_{2DES}+\rho_{Ti}}{2b_{j}}$, $R_{m,k}=\frac{\rho_{2DES}+\rho_{Ti}}{2c_{k}}$. Here, *R_q_=h/e^2^* is the quantum resistance and $k_{F}=\sqrt{2\pi n}$ are the Fermi wave vector, with *n* being the carrier density, $a_{i}$, $b_{j}$, and $c_{k}$ are the contact radius of each point-contact channel in the ballistic regime, the intermediate regime, and the thermal regime, respectively. Previous studies have shown that $R_{m,k}<R_{w,j}< R_{sh,i}$ in the same junction materials because of the decreasing contact radius required to access corresponding regimes [1,9]. It is then straightforward to establish: $\frac{1}{R_{jun}}>\sum_{k} \frac{1}{R_{m,k}}=\frac{\Sigma_{k}2c_{k}}{\rho_{2DES}+\rho_{Ti}}$. This inequality dictates that the total contact radius of the junctions in the thermal regime: $c={\Sigma_{k}c}_{k} < \frac{\rho_{2DES}+\rho_{Ti}}{2R_{jun}}$.

The sheet resistances of Ti and 2DES in their normal states are 0.65 Ω/□ and 384 Ω/□ (*V*_G_ = 0 V), respectively (see Fig. 1d and 1e). The thicknesses of Ti and 2DES are 70 nm and 3.9 nm (details provided in Supplementary Note 6), respectively. Thus, the resistivities of 2DES ($\rho_{2DES}$) and Ti ($\rho_{Ti}$) are estimated to be 1.5 μΩ·m and 0.05 μΩ·m, respectively. The junction resistance $R_{jun}$ is 370 Ω obtained from Fig. 2a. Using these values, the upper bound of the total contact radius *c* is estimated to be 2.1 nm. Moreover, the carrier density *n* is about 1.8 × 10^14^ cm^-2^ derived from Supplementary Fig. 4. The mean free path *l* is thus estimated to be 20 nm, calculated by *l* = *h*/*e*^2^*k_F_R_S_*, where *R*_S_ is the normal sheet resistance of 2DES. Evidently, the estimated contact radius *c* is significantly smaller than the mean free path *l*, which contradicts the adopted assumption that the junctions operate in the thermal regime where each contact radius $c_{k}$ should be much larger than *l* [9]. Therefore, the contribution of Maxwell’s resistance arising from the heating effect to the overall conduction in our Ti/2DES junction must be negligible.

1. Regarding the two conductance dips, studies have revealed that if these dips are caused by the critical current effect due to heating in the point-contact region, they tend to shift significantly inward in the presence of a magnetic field, as the critical current density is reduced [10-14]. In sharp contrast, our results show that the position of the conductance dip remains largely unchanged by the magnetic field (see Supplementary Fig. 10b), as long as the 2DES is in the zero-resistivity state (*B* < 10 T). This indicates that the dip observed in our study does not arise from the critical current effect but rather is an intrinsic characteristic.
2. Based on the above discussion, the critical current/heating effect caused by metallic transport through nonballistic point-contacts has been ruled out. In this case, if the two conductance peaks around *V* = 0 arise from conventional Andreev reflection, then the positions of these peaks should be aligned with, or at least close to, the superconducting gap edges according to the conventional BTK theory [15]. However, the conductance peaks observed in our experiments are positioned approximately at 0.1 meV (see Fig. 2c), a value significantly lower than the superconducting gap of 0.38 meV, as determined by the generalized BCS fit (see Fig. 3f). This suggests that the double conductance peaks featured in our tunneling spectra are not likely due to conventional Andreev reflection.

In summary, all the evidence listed above goes against the trivial, mixed mechanism involving the metallic regime of transport and conventional Andreev reflection, but favors the intrinsic, unified theory proposed in our manuscript invariably.

**Supplementary Note 2. Measurement of Ti/LAO/2DES junction resistance**

At 3 K, where the Ti and the 2DES are both in normal metal states, we measured the large-range tunneling spectrum of the junction (Supplementary Fig. 3), which displays a clear asymmetric tunneling characteristic. Here, a positive bias voltage means that electrons tunnel from the top electrode to the 2DES. By deriving the *I*-*V* curve, we get that the differential resistance near zero bias is 926 Ω. We demonstrate that the measured resistance mainly comes from the Ti/2DES junction, as follows: As shown in Supplementary Fig. 3a, if a current is driven from electrode 3 to 4, the measured nonlocal voltage between 2 and 1 (or 1’) be identical to the voltage between 2 and 5. This implies that the measured nonlocal resistance is equivalent to the junction resistance (*R*_J_), and the measured voltage between 2 and 1 should match the voltage between 2 and 1’. This is consistent with the experimental results (Supplementary Fig. 3b). Therefore, the measured nonlocal resistance primarily originates from the Ti/2DES junction. The validity of this nonlocal method has also been demonstrated in previous studies [6,16-19].

It is important to highlight that the presence of a superconducting state in the Al wire does not impact our findings. This is primarily due to two reasons: First, the role of Al wires in tunneling junctions can be neglected because the measured tunneling resistance is mainly from the Ti/2DES junction, as demonstrated above. Second, the critical magnetic field of superconducting Al is very low, around 10 mT. As shown in Fig. 2b, the key features of the tunneling spectrum (double conductance peaks and double conductance dips) can survive up to more than 10 T, a point at which Al is no longer superconducting.

**Supplementary Note 3. Heuristic analysis of contact-induced Andreev bound states.**

In the model described by Eq. (S1) in Methods, the effect of the metallic lead on the superconductor can be included as a self-energy:

$\sum_{L} (E^{+};\boldsymbol{r},\boldsymbol{r}^{\boldsymbol{'}})=\frac{V^{2}}{2t_{L}}\sum_{\boldsymbol{k}_{\parallel}} \psi_{\boldsymbol{k}_{\parallel}}\left( \boldsymbol{r} \right){\psi_{\boldsymbol{k}_{\parallel}}\left( \boldsymbol{r}^{\boldsymbol{'}} \right)}^{*}e^{-i\gamma\left( E^{+},\boldsymbol{k}_{\parallel} \right)},$ (S7)

$\gamma\left( E^{+},\boldsymbol{k}_{\parallel} \right)=\arccos\frac{E^{+}-(E_{\boldsymbol{k}_{\parallel}}+2t_{L}-\mu_{L})}{2t_{L}},$ (S8)

where $V$ stands for the tunneling rate between the lead and the superconductor ($H_{T}\propto V$); $t_{L}$ is the hopping parameter in the lead; $\boldsymbol{k}_{\parallel}$, $\psi_{\boldsymbol{k}_{\parallel}}\left( \boldsymbol{r} \right)$ and $E_{\boldsymbol{k}_{\parallel}}$ are the wave-vector parallel to the interface and its associated wave-function and eigen-energy in the lead, respectively; $\mu_{L}$ is the chemical potential in the lead. Note that because of its open boundaries in the dimensions parallel to the interface, $\psi_{\boldsymbol{k}_{\parallel}}\left( \boldsymbol{r} \right)$ is a standing wave and the sum over $\boldsymbol{k}_{\parallel}$ can be redundant (i.e., the contributions from $\boldsymbol{k}_{\parallel}$ and ${-\boldsymbol{k}}_{\parallel}$ are equal).

As a heuristic analysis, here we will illustrate the physics in one dimension (1D) for simplicity and leave the fully developed theory to a follow-up paper that is under preparation. In 1D, Eq. (S7) becomes

$\sum_{L} (E^{+};x,x^{'})=\frac{V^{2}}{t_{L}W}\sum_{q} \sin\left( qx \right)sin(qx^{'})e^{-i\gamma\left( E^{+},q \right)},$ (S9)

where $\psi_{q=\frac{n\pi}{W}}\left( x \right)=\sqrt{2/W}sin(qx)$ is a standing wave normalized in $x\in[0,W]$. By Fourier transformation of the above expression, we obtain

$\sum_{L} (E^{+};k,k^{'})=\int_{0}^{W} \frac{dx}{\sqrt{W}}e^{-ikx}\int_{0}^{W} \frac{dx^{'}}{\sqrt{W}}e^{-ik^{'}x^{'}}\sum_{L} (E^{+};x,x^{'})$ (S10a)

$=\frac{V^{2}}{2t_{L}}e^{-i\gamma\left( E^{+},k \right)}[\delta\left( k-k^{'} \right)-\delta\left( k+k^{'} \right)]$, (S10b)

where we have assumed the large $W$ limit and $E_{q}=E_{-q}$ (such that $\gamma\left( E^{+},q \right)= \gamma\left( E^{+},-q \right)$). We immediately see that, in the *k*-space, the self-energy $\sum_{L} (E^{+};k,k^{'})$ not only contains diagonal corrections (proportional to $\delta\left( k-k^{'} \right)$), but also induces scattering between opposite momenta (proportional to $\delta\left( k+k^{'} \right)$) which plays different roles in superconductors of different forms of pairing, as we proceed to show.

Let us compare explicitly the *s*-wave and the *p*-wave cases. Their Hamiltonians (in 1D and in the continuous limit) generically reads

$H_{sSC}\left( k \right)=\xi_{k}\tau_{z}+\Delta_{s}\tau_{x},$ (S11a)

$H_{pSC}\left( k \right)=\xi_{k}\tau_{z}+\Delta_{p}k\tau_{x},$ (S11b)

where $\xi_{k}=\xi_{-k}$ is the normal-state energy, $\tau_{x,y,z}$ are the Pauli matrices associated with the Nambu basis. When the self-energy Eq. (S10b) is included, the two sectors for $k$ and $-k$ will be coupled such that the Green function for the superconductor is given by

$G_{SC}\left( E^{+},k \right)={[E^{+}-\tilde{H}_{SC}\left( k \right)]}^{-1}$, (S12a)

$\tilde{H}_{SC}\left( k \right)=\left( \begin{matrix} H_{SC}\left( k \right)+\epsilon_{L}\left( k \right)\tau_{z}-i\Gamma(k)\tau_{0} & {-\epsilon}_{L}\left( k \right)\tau_{z}+i\Gamma(k)\tau_{0} \\ {-\epsilon}_{L}\left( k \right)\tau_{z}+i\Gamma(k)\tau_{0} & H_{SC}\left( -k \right)+\epsilon_{L}\left( k \right)\tau_{z}-i\Gamma(k)\tau_{0} \end{matrix} \right)$, (S12b)

where $\epsilon_{L}\left( k \right)=\frac{V^{2}}{2t_{L}}\cos\gamma\left( 0,k \right)=\frac{V^{2}}{4{t_{L}}^{2}}\left( \mu_{L}-E_{k}-2t_{L} \right)$ and$\Gamma\left( k \right)=\frac{V^{2}}{2t_{L}}\sin\gamma\left( 0,k \right)=\frac{V^{2}}{4{t_{L}}^{2}}\sqrt{4{t_{L}}^{2}-\left( \mu_{L}-E_{k}-2t_{L} \right)^{2}}>0$ are the real and the imaginary parts of the self-energy around the lead Fermi energy ($E^{+}\simeq0$), respectively.

When the superconductivity is *s*-wave (Hamiltonian is given by Eq. (S11a)), the pairing term $\Delta_{s}\tau_{x}\oplus\tau_{x}\equiv\Delta_{s}\tau_{x}\otimes\sigma_{0}$ (we will use $\sigma_{0,x,y,z}$ as notations for the Pauli matrices associated with the $\pm k$ sectors) anti-commutes with the hermitian part of the self-energy, therefore no in-gap states will be induced. This can be seen explicitly by replacing $H_{SC}\left( k \right)$ in Eq. (S12b) with $H_{sSC}\left( k \right)$ in Eq. (S11a), and obtaining the eigenvalues of $\tilde{H}_{SC}\left( k \right)$:

$\tilde{\varepsilon}_{1}=\sqrt{\xi^{2}+\Delta_{s}^{2}},$ (S13a)

$\tilde{\varepsilon}_{2}=\sqrt{{(\xi+2\epsilon_{L})}^{2}+\Delta_{s}^{2}}-2i\Gamma,$ (S13b)

$\tilde{\varepsilon}_{1}^{'}={-\tilde{\varepsilon}}_{1}^{*}=-\sqrt{\xi^{2}+\Delta_{s}^{2}},$ (S13c)

$\tilde{\varepsilon}_{2}^{'}={-\tilde{\varepsilon}}_{2}^{*} =-\sqrt{\left( \xi+2\epsilon_{L} \right)^{2}+\Delta_{s}^{2}}-2i\Gamma.$ (S13d)

When the superconductivity is *p*-wave (Hamiltonian is given by Eq. (S11b)), in contrast, the pairing term $\Delta_{p}(k\tau_{x})\oplus(-k\tau_{x})\equiv\Delta_{p}k\tau_{x}\otimes\sigma_{z}$ commutes with the self-energy contribution $-\epsilon_{L}\tau_{z}\otimes\sigma_{x}$ which enters through scattering between $\pm k$, therefore in-gap Andreev states can be induced. This can be seen explicitly by replacing $H_{SC}\left( k \right)$ in Eq. (S12b) with $H_{pSC}\left( k \right)$ in Eq. (S11b), and obtaining the eigenvalues of $\tilde{H}_{SC}\left( k \right)$ as follows:

$\tilde{\varepsilon}_{1}=\sqrt{{(\xi+\epsilon_{L}-i\Gamma)}^{2}+{(\Delta_{p}k)}^{2}}+\epsilon_{L}-i\Gamma$, (S14a)

$\tilde{\varepsilon}_{2}=\sqrt{{(\xi+\epsilon_{L}+i\Gamma)}^{2}+{(\Delta_{p}k)}^{2}}-\epsilon_{L}-i\Gamma$, (S14b)

$\tilde{\varepsilon}_{1}^{'}={-\tilde{\varepsilon}}_{1}^{*}=-\sqrt{{(\xi+\epsilon_{L}+i\Gamma)}^{2}+{(\Delta_{p}k)}^{2}}-\epsilon_{L}-i\Gamma$, (S14c)

$\tilde{\varepsilon}_{2}^{'}={-\tilde{\varepsilon}}_{2}^{*}=-\sqrt{{(\xi+\epsilon_{L}-i\Gamma)}^{2}+{(\Delta_{p}k)}^{2}}+\epsilon_{L}-i\Gamma$. (S14d)

At the Fermi surface $\left( k=k_{F} \right), \xi_{k_{F}}=0$ by definition and $\tilde{\varepsilon}_{1}\tilde{\varepsilon}_{2}^{'}=\tilde{\varepsilon}_{2}\tilde{\varepsilon}_{1}^{'}=-\left( \Delta_{p}k_{F} \right)^{2}$. We immediately see that there will be in-gap states corresponding to those $\tilde{\varepsilon}$’s with $|Re\left( \tilde{\varepsilon} \right)|<|\Delta_{p}k_{F}|$.

**Supplementary Note 4. Fit of superconducting energy gap.**

Based on the Dynes model, the experiment tunneling spectra curve d*I*/d*V*(*V*) can be fitted with tunneling current [20]:

$$I\left( V \right)=\frac{1}{2\pi}\int_{0}^{2\pi} d\theta\int_{-\infty}^{+\infty} d\varepsilon\left[ f\left( \varepsilon\right)-f\left( \varepsilon+eV \right) \right]\cdot\mathrm{Re} \left( \frac{\varepsilon+eV+i\Gamma}{\sqrt{\left( \varepsilon+eV+i\Gamma\right)^{2}-\Delta\left( \theta\right)^{2}}} \right),$$

where$f(\varepsilon)$ is the Fermi distribution function containing the information of temperature, $\Gamma$ is the broadening parameter, $\Delta(\theta)$ is the superconducting gap, $\theta$ is the azimuth angle along the Fermi surface in the Brillouin zone.For *s*-wave gap: $\Delta(\theta)=\Delta_{0}$, and the proposed *p*-wave gap: $\Delta(\theta)=\Delta_{0}\left| k_{x}+ik_{y} \right|$. Therefore, the derived gap sizes are similar for the *s*-wave and *p*-wave gap functions, which we collectively refer to as the “full gap”. For *d*-wave superconductors, $\Delta(\theta)=\Delta_{0}\left( 2k_{x}k_{y} \right)$. The best fits to the data are shown in Fig. 3e and Supplementary Fig. 7a. Temperature-dependent *Δ* and *Γ* extracted from the fits are shown in Fig. 3f and Supplementary Fig. 7b. Error bars are estimated from the standard deviation (s.d.) of the fit. The black line is the generalized BCS model fit [21-23] with *T*_C_ = 2.05 K and 2*Δ*(*T* = 0)/*k*_B_*T*_C_ = 4.3. The temperature-dependent 2DES resistance indicates a superconducting transition temperature of 2.06 K (Supplementary Fig. 7c), which is consistent with the fit result.

**Supplementary Note 5. Discussion on different sample growth temperatures**

In a control experiment, we grew LAO at room temperature under an O_2_ pressure of 1×10^-4^ mbar. As depicted in Supplementary Fig. 8, the superconducting transition temperature is now only 0.61 K, which is much lower than 1.38 K observed in samples grown at 620 ℃; To access a broad temperature window for superconducting LAO/KTO interface, we opted for the samples grown at 620 ℃ for the tunneling measurements. Contrasted with room temperature growth, a growth temperature of 620 ℃ may introduce a higher number of oxygen vacancies during the deposition of the LAO film. This would increase the native carrier density and hence raise the superconducting transition temperature [24].

**Supplementary Note 6. Determination of the superconducting 2DES thickness**

Supplementary Fig. 9a and 9b show the normalized magnetoresistances measured in magnetic fields aligned parallel and perpendicular to the LAO/KTO(111) interface, respectively. The upper critical magnetic fields, defined at half of *R*_N_, are summarized in Supplementary Fig. 9c. The temperature dependence of $\text{B}_{\text{C}\perp}$ is well fitted by the Ginzburg Landau theory [25]: $\text{B}_{\text{C}\perp}\text{(}\text{T}\text{)}\text{ }\text{=}\text{ }\text{Φ}_{\text{0}}\left( \text{1-}\text{T}\text{/}\text{T}_{\text{C}} \right)\text{/}\left[ \text{2π}\left( \text{ξ}_{\text{GL}} \right)^{\text{2}} \right]$, where $\text{Φ}_{\text{0}}$ is the flux quantum and $\text{ξ}_{\text{GL}}$ is the Ginzburg Landau coherence length at zero temperature, estimated to be 18.6 nm from this fit. For a 2D superconductor, the temperature behavior of *B*_C||_ follows the Tinkham’s model [26], described as $\text{B}_{\text{C||}}\text{(}\text{T}\text{)}\text{ }\text{=}\text{ }\text{Φ}_{\text{0}}\left[ \text{12}\left( \text{1-}\text{T}\text{/}\text{T}_{\text{C}} \right) \right]^{\text{1/2}}\text{/}\left( \text{2π}\text{d}\text{ξ}_{\text{GL}} \right)$, where *d* is the superconducting 2DES thickness. By fitting to *B*_C||_(*T*), we obtained *d* = 3.9 nm, which is consistent the previous study on the LAO/KTO(111) interface [27].

1. **Figures and Table**


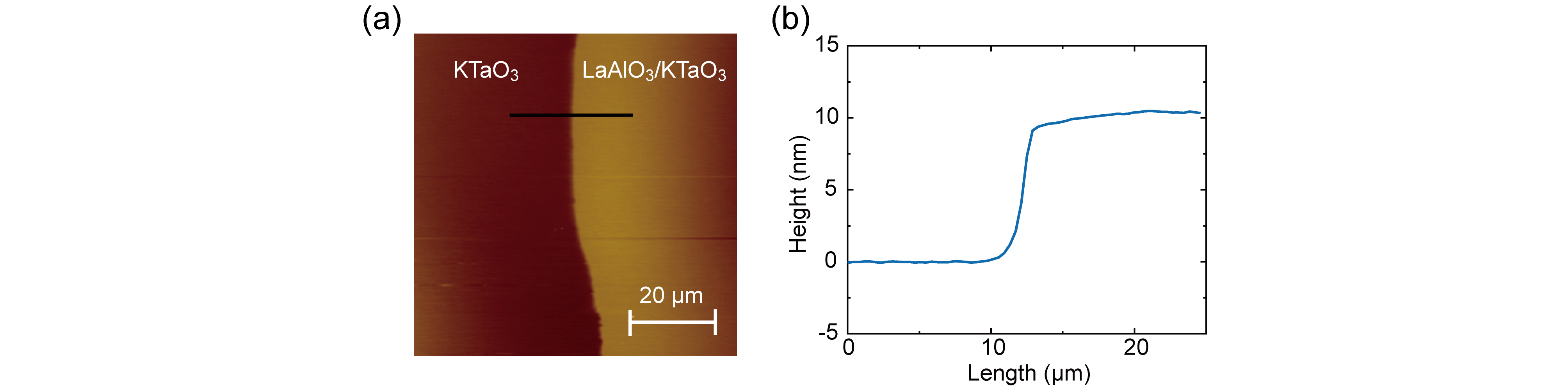


**Supplementary Figure 1.** Growth rate calibration. (a) Atomic force microscopy image of the boundary between KTO substrate and LAO/KTO. The LAO on the right side of the boundary is grown with 1500 laser pulses. The left side of the boundary is blocked by a mask during growth. (b) Height profile along the black line in (a) gives a thickness of ~10 nm. The LAO growth rate calibrated by atomic force microscopy is ~0.0067 nm/pulse.


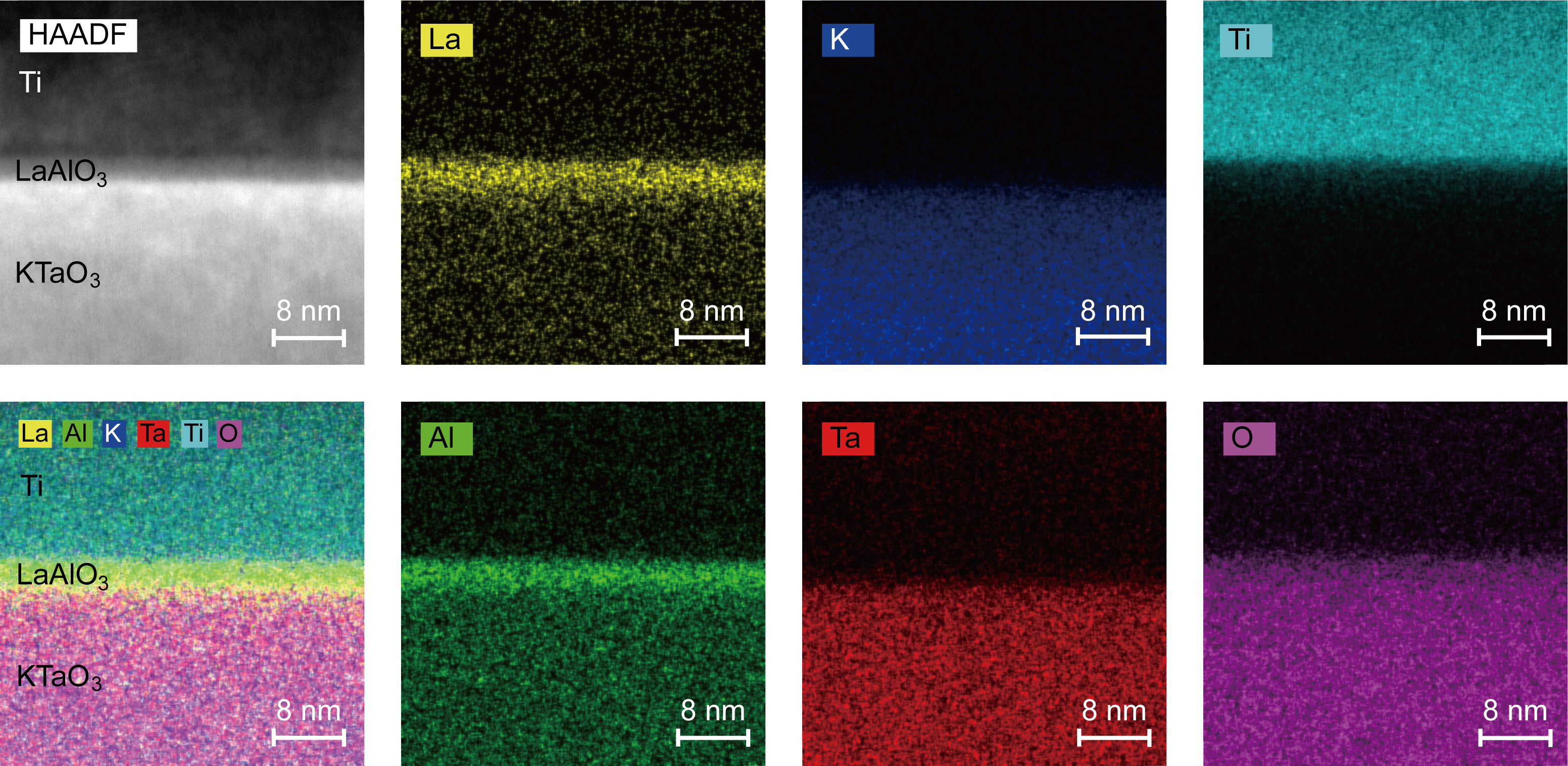


**Supplementary Figure 2.** HAADF-STEM image and corresponding EDS elemental mapping of Ti/LAO/KTO heterostructure. EDS elemental mapping shows that the device is clearly resolved structurally and chemically. The LAO growth rate calibrated by STEM (~0.0067 nm/pulse) is consistent with that by atomic force microscopy.


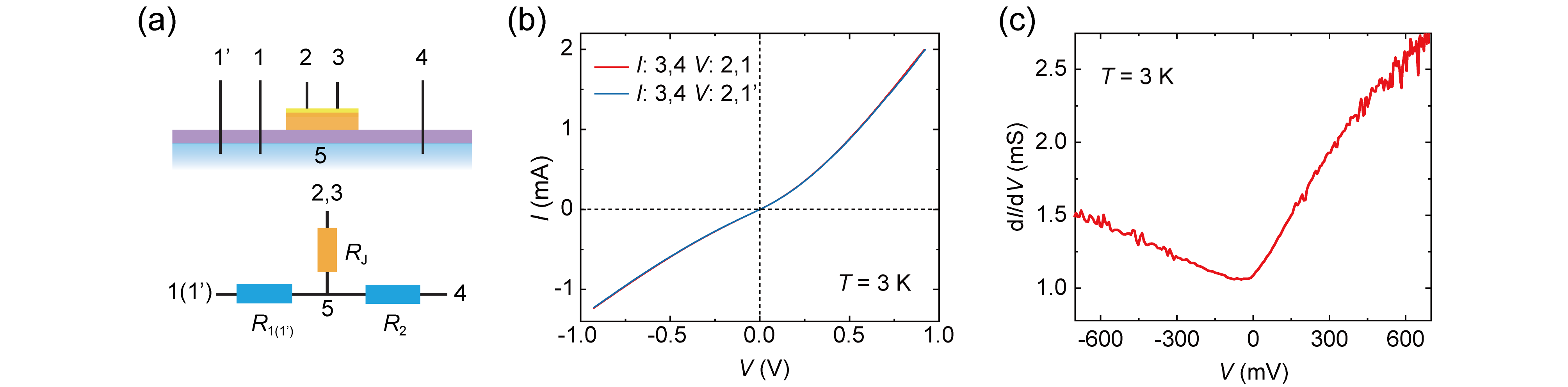


**Supplementary Figure 3.** Tunneling measurements of the Ti/LAO/2DES junction. (a) Schematic of the device. Here electrodes 1, 1’ and 4 connect directly to the 2DES, while electrodes 2 and 3 connect to the Ti/Au pads on top of the device. Below is the equivalent circuit of the device. (b) Large-range current-voltage (*I*-*V*) tunneling characteristic measured at *T* = 3 K, where the Ti and the 2DES are both in normal metal state. A positive current is provided by electrons tunneling from the Ti into the 2DES. (c) Experimental differential conductance, d*I*/d*V*(*V*), derived from (b). The data were measured in Sample #1.


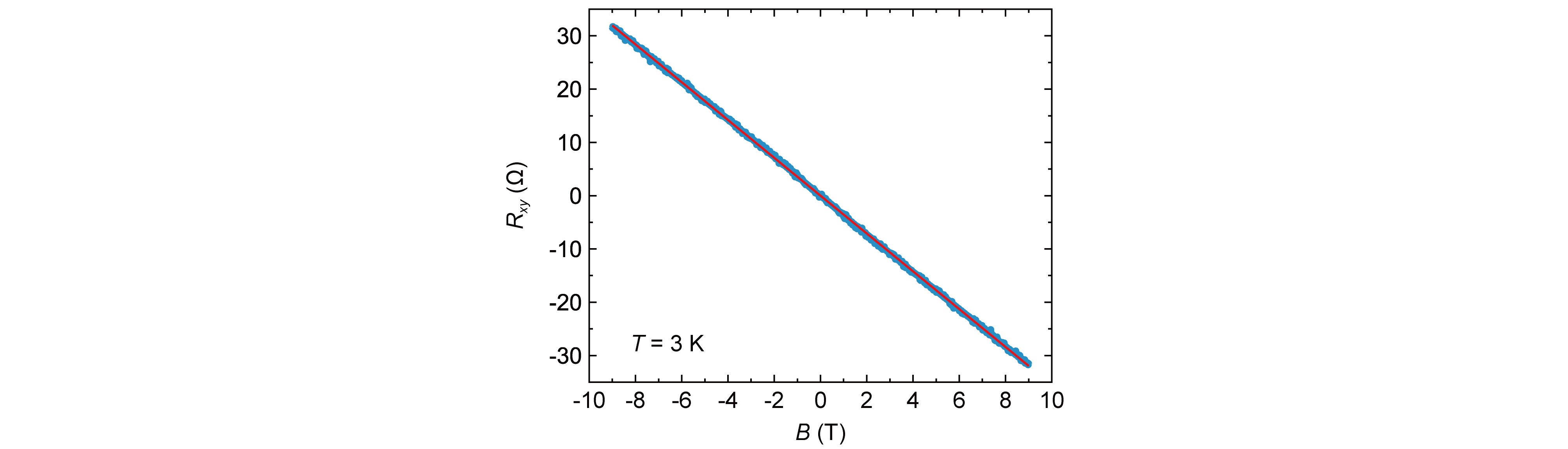


**Supplementary Figure 4.** Carrier density characterization of the LAO/KTO(111) interface. Magnetic field (*B*) dependence of Hall resistance (*R_xy_*) was measured at *T* = 3 K, resulting in the carrier density (*n*) of 1.8 × 10^14^ cm^-2^, calculated using the formula *R_xy_* = -*B*/*ne*, where *e* is the electron charge. The red line represents the linear fit.
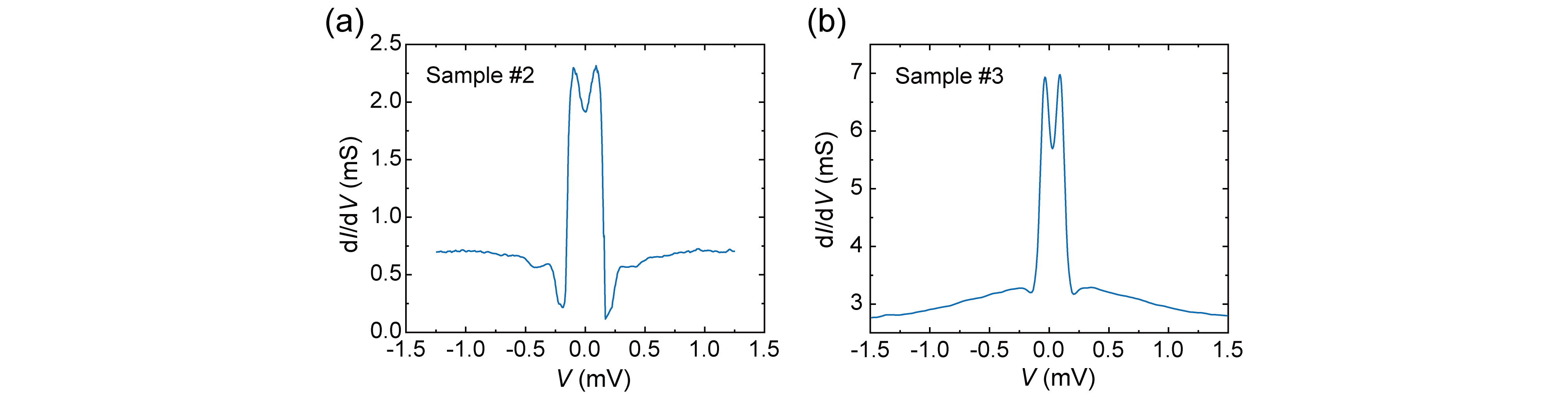


**Supplementary Figure 5.** Tunneling spectra of the other *d*_LAO_ = 3.2 nm Ti/LAO/KTO(111) samples. (a) Tunneling spectrum of Sample #2 at *V*_G_ = -160 V and *T* = 0.5 K. (b) Tunneling spectrum of Sample #3 at *V*_G_ = -160 V, *T* = 0.05 K and *B*_||_ = 0.8 T. The role of the small parallel magnetic field here is to kill the superconductivity of Ti at *T* = 0.05 K, while the superconductivity of the 2DES still survives. Pronounced symmetrical double peaks and double dips can be observed in Sample #2 and Sample #3, similar to the result of Sample #1.

**
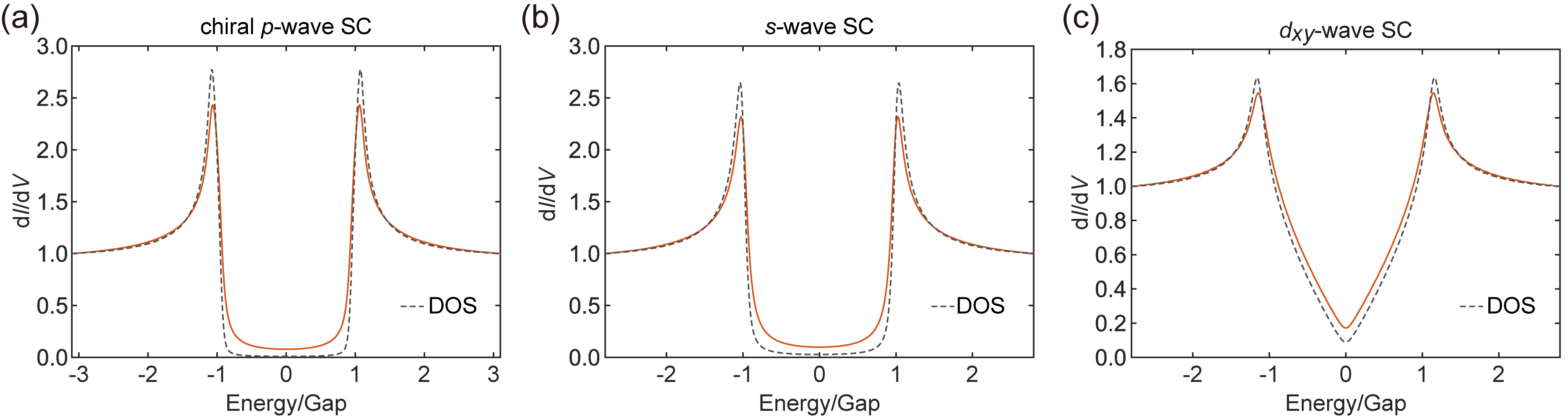
**

**Supplementary Figure 6.** Calculated tunneling spectra for weak metal-superconductor coupling. (a-c) Differential conductance data (normalized by the 2DES normal state value) in red lines for generic chiral *p*-wave, *s*-wave, and *d_xy_*-wave superconductors, respectively. The normalized DOS for a pristine superconductor in each case is also plotted (in black dashed lines) for reference. In these calculations, we have used the same parameters as in Fig. 3(b-d) except that $t_{L}=1$ and $V=0.1$ in all cases here, which is representative in the weak contact coupling regime (cf. the parameters given in Supplementary Table 1).


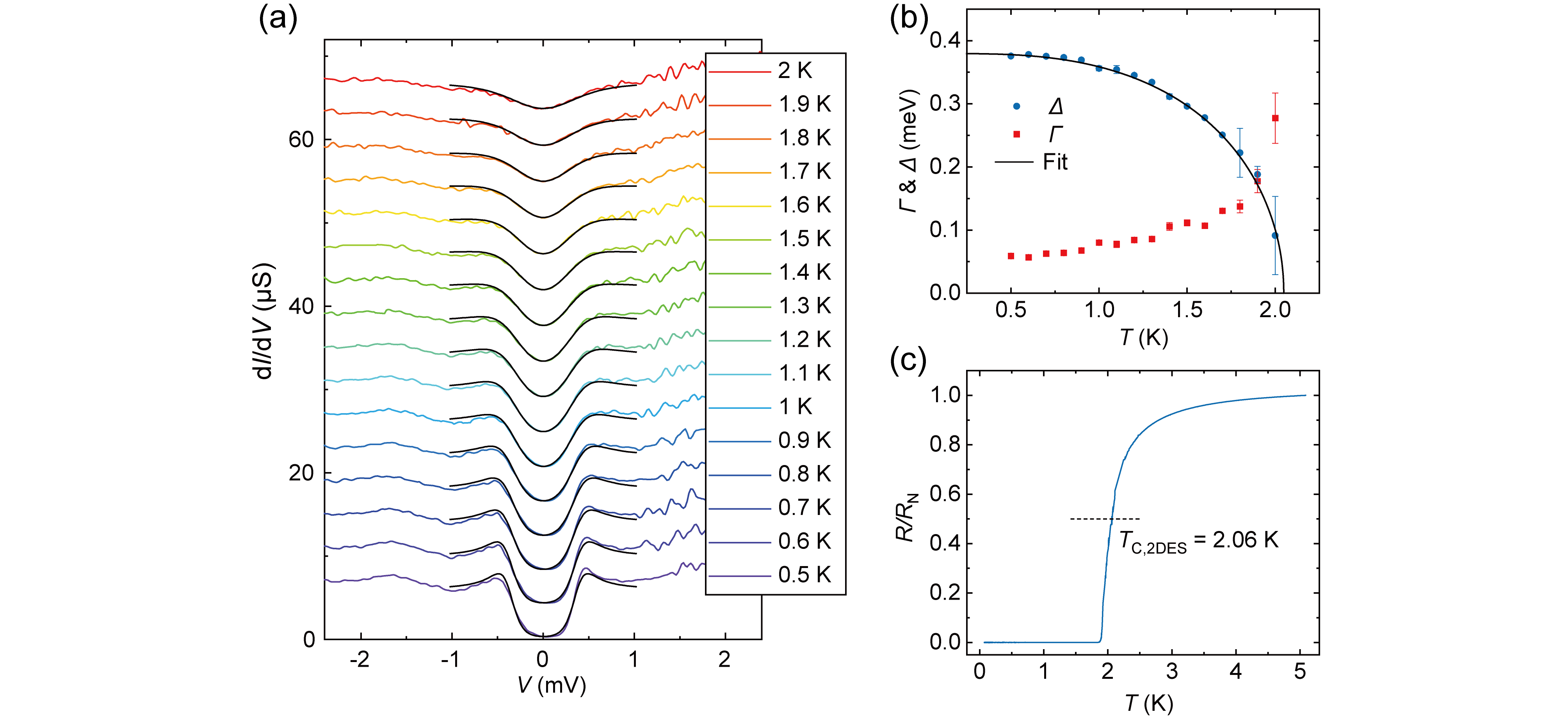


**Supplementary Figure 7.** Temperature-dependent U-shaped superconducting gap of the Ti/LAO/KTO(111) device with *d*_LAO_ = 4 nm. (a) Tunneling spectra at different temperature. The data here is symmetrized from the raw data. The black lines are the Dynes model fits with full gap function. (b) Temperature dependence of *Δ* and *Γ* extracted from the fits. Error bars are estimated from the standard deviation (s.d.) of the fit. The black line is the BCS model fit with *T*_C_ = 2.05 K and 2*Δ*(*T* = 0)/*k*_B_*T*_C_ = 4.3. (c) Temperature-dependent 2DES normalized resistance indicates a superconducting transition temperature of 2.06 K, which is consistent with the result of (b). All measurements were performed in Sample #4.


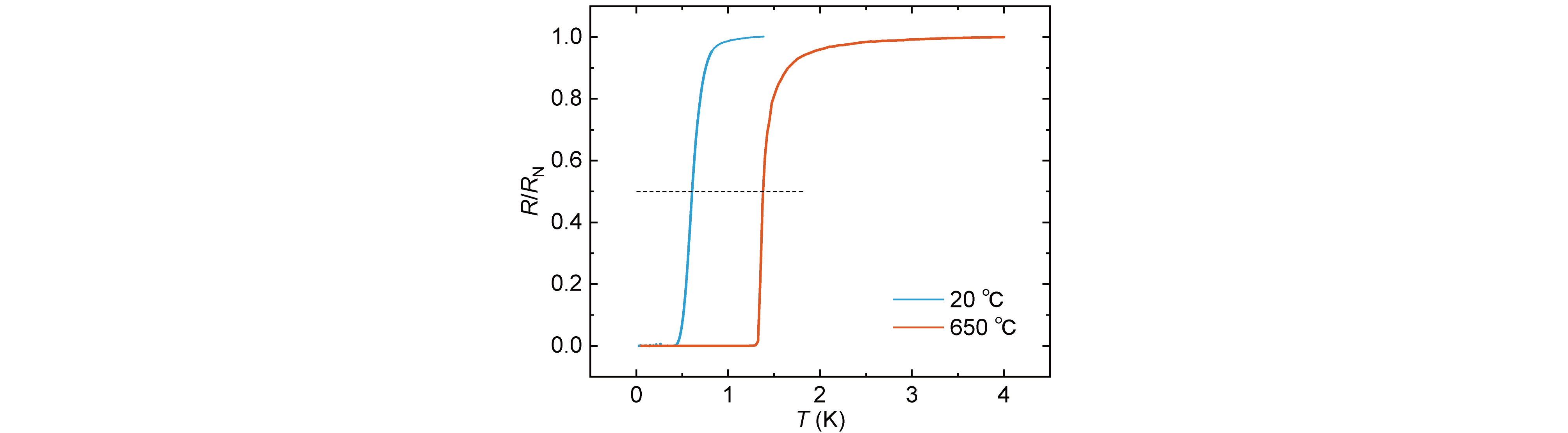


**Supplementary Figure 8.** Superconductivity of samples grown at different temperatures. Normalized resistance as a function of temperature for LAO/KTO samples grown at 620 ℃ and room temperature, respectively.


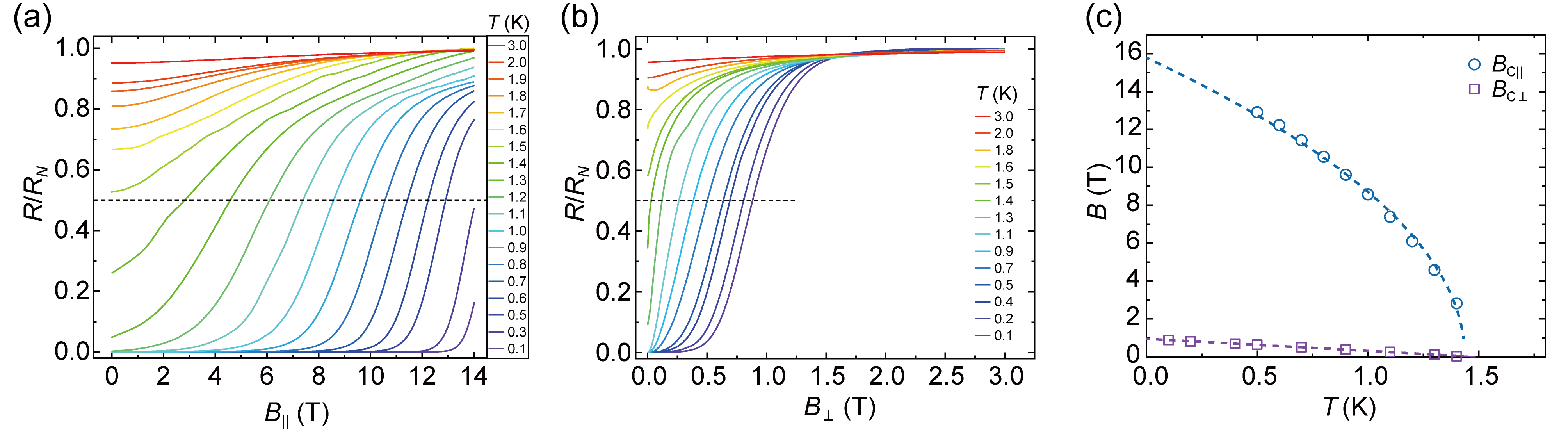


**Supplementary Figure 9.** Superconducting upper critical field of the LAO/KTO(111) interface. (a, b) Normalized resistance measured at different temperatures as a function of the parallel and perpendicular magnetic fields, respectively. (c) Temperature dependence of critical fields, which are determined at half of *R*_N_. The dashed lines show the theoretical fits (cf. Supplementary Note 6). All measurements were performed in Sample #1.


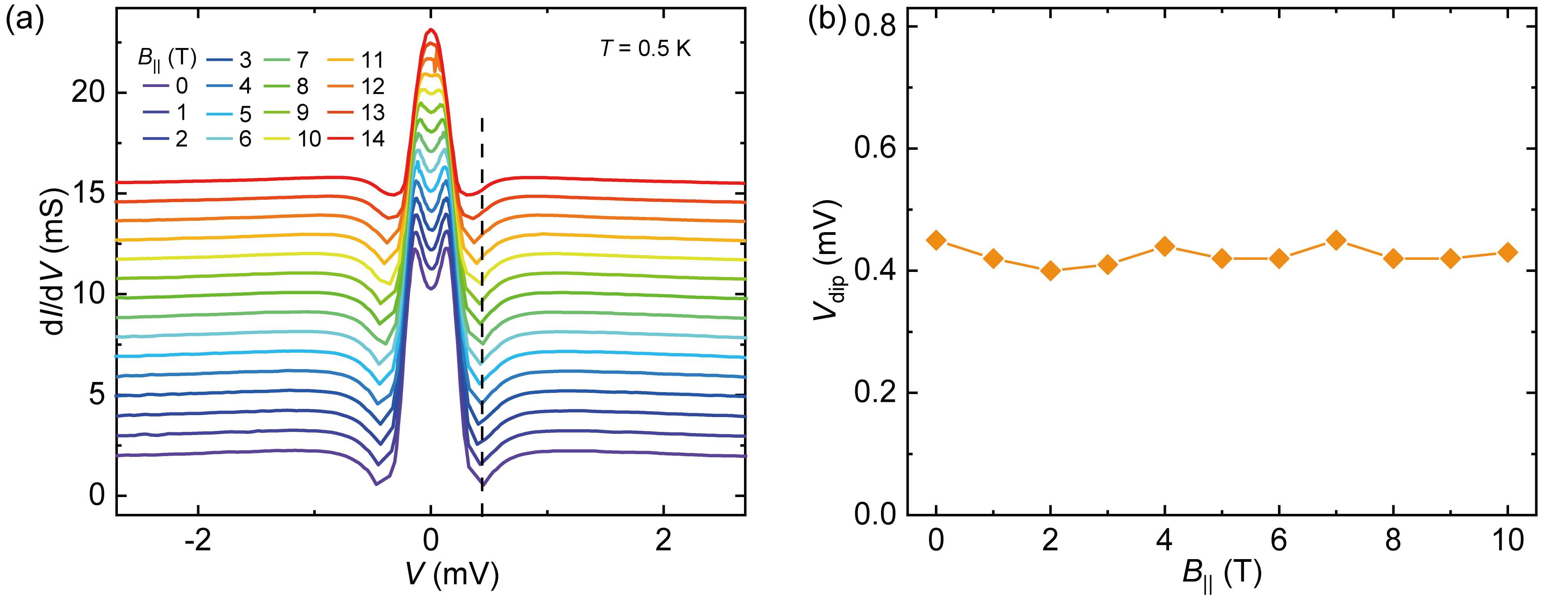


**Supplementary Figure 10.** Magnetic field-dependent tunneling spectra of the Ti/LAO/KTO junction. (a) Tunneling spectra at different parallel magnetic fields with *T* = 0.5 K. The curves are shifted vertically for clarity. (b) Positions of the conductance dips in (a) versus the parallel magnetic field. All measurements were performed in Sample #1 with *V*_G_ = -160 V.

**Supplementary Table 1.** Parameters of numerical simulations

| common | | $\boldsymbol{s}$-wave SC | | | $\boldsymbol{p}$-wave SC | | | $\boldsymbol{d}_{\boldsymbol{xy}}$-wave SC | | |
| --- | --- | --- | --- | --- | --- | --- | --- | --- | --- | --- |
| $\boldsymbol{\mu}$ | $\mu_{L}$ | $\Delta_{s}$ | $t_{L}$ | $V$ | $\Delta_{p}$ | $t_{L}$ | $V$ | $\Delta_{d_{xy}}$ | $t_{L}$ | $V$ |
| 1.4 | 3.4 | 0.1 | 0.5 | 5.0 | 0.1 | 0.7 | 5.0 | 0.1 | 1.0 | 2.0 |

**References**

1. Sheet G, Mukhopadhyay S, Raychaudhuri P. Role of critical current on the point-contact Andreev reflection spectra between a normal metal and a superconductor. *Phys Rev B* 2004; **69**: 134507.

2. Duif AM, Jansen AGM, Wyder P. Point-Contact Spectroscopy. *J Phys Condens Matter* 1989; **1**: 3157-3189.

3. Srikanth H, Raychaudhuri AK. Transition from Metallic to Tunneling-Type Conductance in Metal-Metal and Normal-Metal Superconductor Point Contacts. *Phys Rev B* 1992; **46**: 14713-14719.

4. Frederiksen T, Lorente N, Paulsson M, Brandbyge M. From tunneling to contact: Inelastic signals in an atomic gold junction from first principles. *Phys Rev B* 2007; **75**: 235441.

5. Wolf EL. *Principles of electron tunneling spectroscopy*: Oxford University Press, 1985.

6. Richter C, Boschker H, Dietsche W *et al.* Interface superconductor with gap behaviour like a high-temperature superconductor. *Nature* 2013; **502**: 528-531.

7. Moodera JS, Kinder LR, Wong TM, Meservey R. Large Magnetoresistance at Room Temperature in Ferromagnetic Thin Film Tunnel Junctions. *Phys Rev Lett* 1995; **74**: 3273-3276.

8. Russo S, Klapwijk TM, Schoch W, Limmer W. Correlation effects in the density of states of annealedGa_1−_*_x_*Mn*_x_*As. *Phys Rev B* 2007; **75**: 033308.

9. Daghero D, Gonnelli RS. Probing multiband superconductivity by point-contact spectroscopy. *Supercond Sci Technol* 2010; **23**: 043001.

10. Sasaki S, Kriener M, Segawa K *et al.* Topological Superconductivity in Cu*_x_*Bi_2_Se_3_. *Phys Rev Lett* 2011; **107**: 217001.

11. Sasaki S, Ren Z, Taskin AA *et al.* Odd-parity pairing and topological superconductivity in a strongly spin-orbit coupled semiconductor. *Phys Rev Lett* 2012; **109**: 217004.

12. Kirzhner T, Lahoud E, Chaska KB *et al.* Point-contact spectroscopy of Cu_0.2_Bi_2_Se_3_ single crystals. *Phys Rev B* 2012; **86**: 064517

13. Aggarwal L, Gayen S, Das S *et al.* Mesoscopic superconductivity and high spin polarization coexisting at metallic point contacts on Weyl semimetal TaAs. *Nat Commun* 2017; **8**: 1348.

14. Kurter C, Finck ADK, Huemiller ED *et al.* Conductance Spectroscopy of Exfoliated Thin Flakes of Nb*_x_*Bi_2_Se_3_. *Nano Lett* 2019; **19**: 38-45.

15. Blonder GE, Tinkham M, Klapwijk TM. Transition from metallic to tunneling regimes in superconducting microconstrictions: Excess current, charge imbalance, and supercurrent conversion. *Phys Rev B* 1982; **25**: 4515-4532.

16. Swartz AG, Cheung AKC, Yoon H *et al.* Superconducting Tunneling Spectroscopy of Spin-Orbit Coupling and Orbital Depairing in Nb:SrTiO_3_. *Phys Rev Lett* 2018; **121**: 167003.

17. Swartz AG, Inoue H, Merz TA *et al.* Polaronic behavior in a weak-coupling superconductor. *Proc Natl Acad Sci* 2018; **115**: 1475-1480.

18. Yan YD, Guo LH, Li L *et al.* Tuning the Kondo effect via gating-controlled orbital selection in the LaAlO_3_/SrTiO_3_ interfacial *d*-electron system. *Phys Rev B* 2020; **101**: 035119.

19. Guo L, Yan Y, Xu R *et al.* Zero-Bias Conductance Peaks Effectively Tuned by Gating-Controlled Rashba Spin-Orbit Coupling. *Phys Rev Lett* 2021; **126**: 057701.

20. Dynes RC, Garno JP, Hertel GB, Orlando TP. Tunneling Study of Superconductivity near the Metal-Insulator-Transition. *Phys Rev Lett* 1984; **53**: 2437-2440.

21. Bardeen J, Cooper LN, Schrieffer JR. Theory of Superconductivity. *Phys Rev* 1957; **108**: 1175-1204.

22. Bardeen J, Cooper LN, Schrieffer JR. Microscopic Theory of Superconductivity. *Phys Rev* 1957; **106**: 162-164.

23. Millán JS, Pérez LA, Wang C. *p*-wave superconductivity in a two-dimensional generalized Hubbard model. *Phys Lett A* 2005; **335**: 505-511.

24. Moon SY, Moon CW, Chang HJ *et al.* Comprehensive study on critical role of surface oxygen vacancies for 2DEG formation and annihilation in LaAlO_3_/SrTiO_3_ heterointerfaces. *Electron Mater Lett* 2016; **12**: 243-250.

25. Kozuka Y, Kim M, Bell C *et al.* Two-dimensional normal-state quantum oscillations in a superconducting heterostructure. *Nature* 2009; **462**: 487-490.

26. Tinkham M. Effect of Fluxoid Quantization on Transitions of Superconducting Films. *Phys Rev* 1963; **129**: 2413.

27. Chen Z, Liu Y, Zhang H *et al.* Electric field control of superconductivity at the LaAlO_3_/KTaO_3_(111) interface. *Science* 2021; **372**: 721-724.
